# Combination of DESI2 and IP10 gene therapy significantly improves therapeutic efficacy against murine carcinoma

**DOI:** 10.18632/oncotarget.17623

**Published:** 2017-05-05

**Authors:** Chao Lin, HuaYing Yan, Jun Yang, Lei Li, Mei Tang, Xinyu Zhao, Chunlai Nie, Na Luo, Yuquan Wei, Zhu Yuan

**Affiliations:** ^1^ State Key Laboratory of Biotherapy, Collaborative Innovation Center of Biotherapy, West China Hospital, Chengdu, Sichuan University, Chengdu, 610041, China; ^2^ Department of Functional Imaging, Sichuan Provincial Women's and Children's Hospital, Chengdu, 610031, China; ^3^ Nankai University School of Medicine, Collaborative Innovation Center of Biotherapy, Tianjin, 300071, China

**Keywords:** DESI2, IP10, apoptosis, anti-angiogenesis, antitumor immunity

## Abstract

DESI2 (also known as PNAS-4) is a novel pro-apoptotic gene activated during the early response to DNA damage. We previously reported that overexpression of DESI2 induces S phase arrest and apoptosis by activating checkpoint kinases. The present study was designed to test whether combination of DESI2 and IP10 could improve the therapy efficacy *in vitro* and *in vivo*. The recombinant plasmid co-expressing DESI2 and IP10 was encapsulated with DOTAP/Cholesterol nanoparticle. Immunocompetent mice bearing CT26 colon carcinoma and LL2 lung cancer were treated with the complex. We found that, *in vitro*, the combination of DESI2 and IP10 more efficiently inhibited proliferation of CT26, LL2, SKOV3 and A549 cancer cells via apoptosis. *In vivo*, the combined gene therapy more significantly inhibited tumor growth and efficiently prolonged the survival of tumor bearing mice. Mechanistically, the augmented antitumor activity *in vivo* was associated with induction of apoptosis and inhibition of angiogenesis. The anti-angiogenesis was further mimicked by inhibiting proliferation of immortalized HUVEC cells *in vitro*. Meanwhile, the infiltration of lymphocytes also contributed to the enhanced antitumor effects. Depletion of CD8+ T lymphocytes significantly abrogated the antitumor activity, whereas depletion of CD4+ T cells or NK cells showed partial abrogation. Our data suggest that the combined gene therapy of DESI2 and IP10 can significantly enhance the antitumor activity as apoptosis inducer, angiogenesis inhibitor and immune response initiator. The present study may provide a novel and effective method for treating cancer.

## INTRODUCTION

Some anti-cancer strategies, such as tumor suppressor gene re-introduction, anti-angiogenesis and induction of apoptosis etc., have displayed remarkable therapeutic effects [1, 2]. Among these antitumor strategies, apoptosis induction, anti-angiogenesis and antitumor immune response are currently regarded as three promising approaches [3–5].

DESI2, also known as PNAS-4, is a pro-apoptotic gene, and is activated in response to DNA damage [6, 7]. DESI2 overexpression displays strong pro-apoptotic activity on osteosarcoma U2OS cells [6, 7]. The DESI2 molecule has several hydrophobic motifs and one conserved N-terminal DUF (domain of unknown function) 862 domain, which is recently identified to be associated with deubiquitination [7–9]. The amino acid homology of DESI2 among different species is very high [7]. Additionally, DESI2 is a member of the PPPDE superfamily [7, 10]. More importantly, elevated expression of DESI2 could induce apoptosis of many types of cancer cells such as lung and colon cancer cells. Furthermore, it showed strong antitumor activity *in vivo* and enhances the therapeutic efficacy of cisplatin, gemcitabine, honokiol and radiation [7, 11–15]. Mechanistically, DESI2 arrests cells at S-phase and induces apoptosis by activating checkpoint kinases [7, 9, 12]. These observations suggest that DESI2 is a potential candidate for cancer gene therapy.

Chemokines are a family of cytokines [16]. It can induce chemotaxis of cells expressing corresponding chemokine receptors or direct leukocyte migration [17, 18]. The chemokines include four subfamilies, i.e., CX3C, CXC, CC and C subfamilies [19–22]. IP10 (also known as CXCL10) belongs to the CXC subfamily [22]. It specifically activates CXCR3, a G protein-coupled receptor [23, 24], which is mainly expressed on T lymphocytes [25, 26], NK cells [26, 27] and macrophages [26, 28]. IP10 is a multi-function molecule that can inhibit cell proliferation *via* apoptosis and stimulate immunological cells as well as anti-angiogenesis [24, 26]. Currently, due to its multiple anti-tumor effects, IP10 is considered as a condicate target for cancer gene therapy and received more and more attention [2, 29].

Combination therapy with different cytotoxic agents, which specifically interfere with differently key pathways such as controlling cell survival, proliferation, invasion etc., is a promising therapeutic approach [30]. Furthermore, emerging evidences showed that there was a significant improvement in overall survival among patients who received combination therapy [31–33]. These observations promoted us to hypothesize that the induction of apoptosis by DESI2 and the antiangiogenic and immunological stimulating activities of IP10 could work cooperatively to enhance therapeutic efficacy against murine carcinoma. To test the hypothesis, we constructed a recombinant plasmid expressing both DESI2 and IP10, and delivered the plasmid to murine tumors by liposome-encapsulated method as described previously [2]. We investigated the anti-tumor effects of DESI2 combined with IP-10 *in vitro* and *in vivo*, and further elucidated the action mechanism. To our knowledge, we provided experimental evidence for the first time that combination of DESI2 and IP10 could significantly enhances therapeutic efficacy against murine carcinoma.

## RESULTS

### Co-expression of DESI2 and IP10 inhibits growth of cancer cells *in vitro*

**Figure 1:**
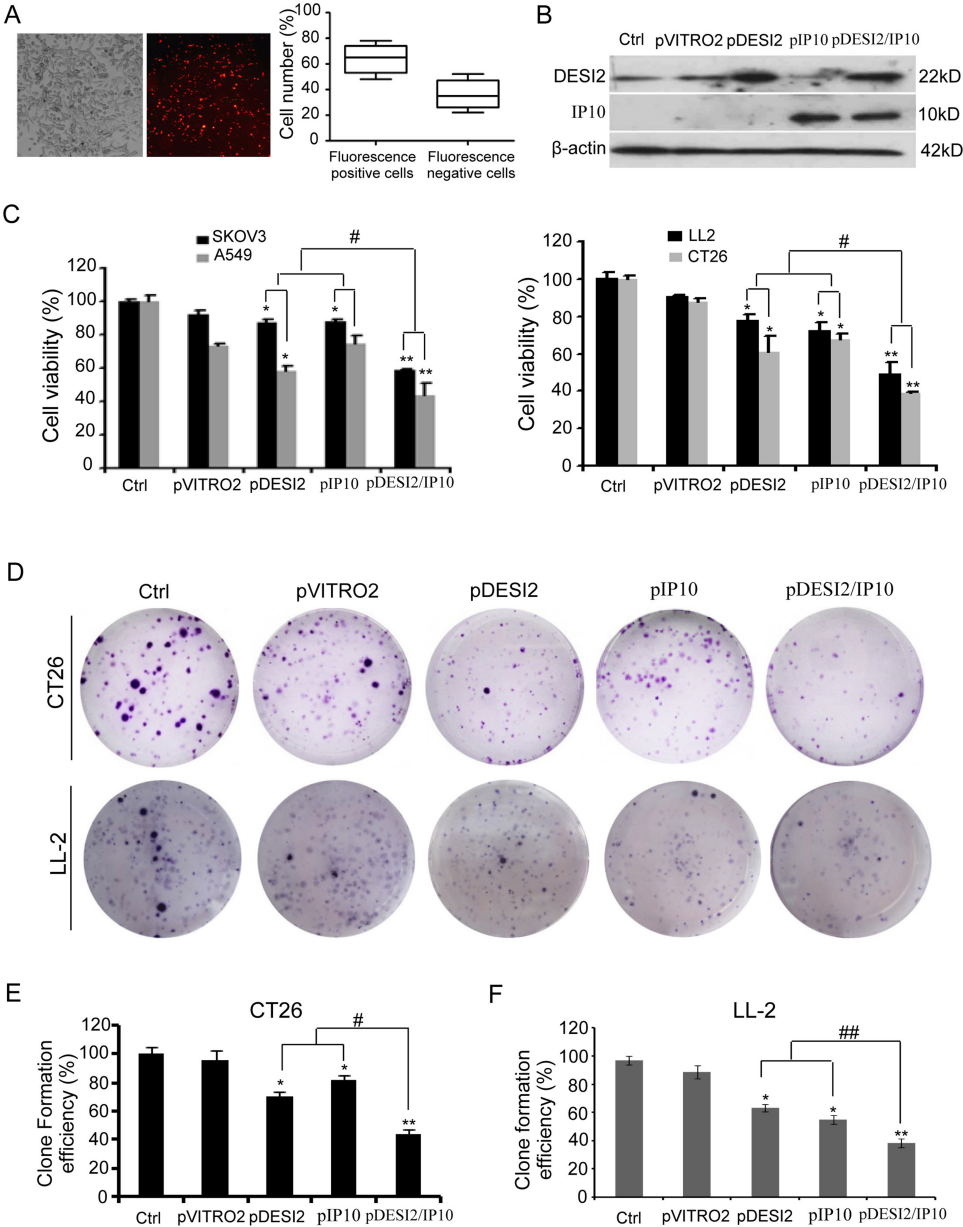
Reduced viability of cancer cells *in vitro* by co-expression of DESI2 and IP10 (**A**) CT26 cells were transfected with pVITRO2-RFP plasmid for 36h, and then the transfection efficiency was estimated by a fluorescence microscope. The representative picture of red fluorescence positive cells was shown (left panel). Transfection efficiency was further quantified as the percentage of fluorescence positive cells to total cells. Bars, SD; columns, mean (*n* = 5) (left panel). (**B**) Western blotting analysis of DESI2 and/or IP10 expression *in vitro* after transfection of CT26 cells. β-actin was used as a loading control. (**C**) Treatment of SKOV3, A549, LL2 and CT26 cells with DESI2 plus IP10 for 48 h reduced their viabilities more significantly than that of DESI2 alone or IP10 alone did. The MTT assay was carried out as described in Materials and Methods. Statistically significant differences compared with the two control groups (*P < 0.05; **P <0.01) and the two single-treatment groups (^#^*P* < 0.05). Percentage of survival was calculated. Bars, SD; columns, mean (*n* = 3). In each experiment, the medium-only treatment (untreated) indicates 100% cell viability. (**D**) Colony-formation assays were further used to evaluate the viability of CT26 and LL2 cells. Co-expression of DESI2 and IP10 resulted in significant inhibition of clone formation compared with the DESI2 and IP10 groups. (**E**) Clone formation efficiency of CT26 cells was quantified as the percentage of different group clones to control clone numbers. Statistically significant differences compared with the two control groups (*P < 0.05; **P <0.01) and the two single-treatment groups (^#^*P* < 0.05). Bars, SD; columns, mean (*n* = 3). (**F**) Clone formation efficiency of LL2 cells was quantified as the percentage of different group clones to control clone numbers. Statistically significant differences compared with the two control groups (*P < 0.05; **P <0.01) and the two single-treatment groups (^##^*P* < 0.01). Bars, SD; columns, mean (*n* = 3).

We first selected LL2, CT26, SKOV3 and A549 cancer cells to test the anti-proliferative efficacy of DESI2 and IP10. Cells were transfected with the following vectors: pVITRO2, pDESI2, pIP10, pDESI2/IP10. About 60% of the cells were transfected as indicated by the RFP signal (Figure 1A). DESI2 overexpression was further verified by western blotting analysis (Figure 1B). After treatment for 48h, MTT assay was used to detect cell viability. Co-expression of DESI2 and IP10 reduced cell viability of SKOV3, A549, LL2 and CT26 cells more effectively than that of pDESI2 or pIP10 alone (Figure 1C). The reduced viability was also observed in LL2 and CT26 cells by colony-formation assays (Figure 1D–1F). To exclude the possibility that our observation is an artifact, we also carried out a parallel experiment to detect the viability of A549 cells after transfection with pVITRO2-RFP plasmid. As expected, no difference in cell viability was observed between pVITRO2-RFP-transfected A549 cells and pVITRO2-transfected A549 cells (data not shown), suggesting that inhibition of cell proliferation is not an artifact of the transfection but by DEIS2 and IP10.

### Induction of apoptosis *in vitro* by co-expression of DESI2 and IP10

**Figure 2:**
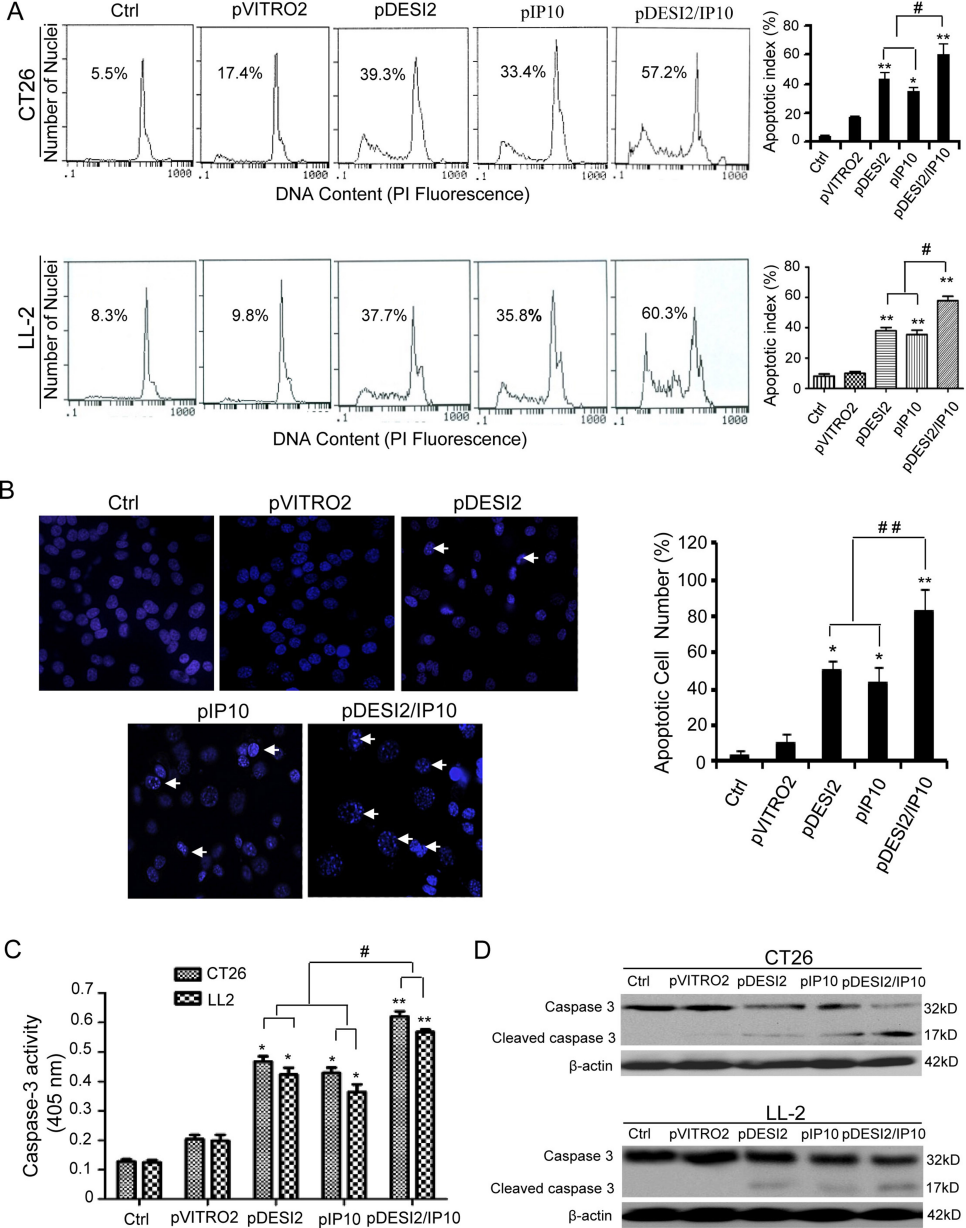
Induction of apoptosis of CT26 tumor cells *in vitro* by DESI2 and/or IP10 (**A**) Representative DNA fluorescence histograms of propidium iodide-stained cells. CT26 and LL-2 cells were transfected with DESI2 and/or IP10 for 48 h. CT26 and LL-2 cells were untreated or transfected with pVITRO2 empty vector , pDESI2, pIP10 or pDESI2/IP10, with (1) 5.5 % and 8.3 %, (2) 17.4 % and 9.8 %, (3) 39.3% and 37.7%, (4) 33.4% and 35.8, and (5) 57.2% and 60.3% sub-G1 cells (apoptotic cells), respectively, as assessed by flow cytometry (left). Co-expression of DESI2 and IP10 induced more significant apoptosis of CT26 and LL2 cells than the DESI2 and IP10 groups did (right). Statistically significant differences compared with the two control groups (*P < 0.05; **P < 0.01) and the two single-treatment groups (^#^*P <* 0.05). Bars, SD; columns, mean (*n* = 3). (**B**) Normal and apoptotic nuclear morphologies of CT26 cells were shown (left). CT26 cells were treated with the same conditions as described above and used to analyze the nuclear morphology by Hoechst 33258 staining. Arrows represented some typically apoptotic nuclear morphology. Co-expression of DESI2 and IP10 resulted in significant increase of the number of the apoptotic nuclei compared with the DESI2 and IP10 groups (right). Bars, SD; columns, mean (*n* = 3, *p < 0.05; **p < 0.01; ^##^*P <* 0.01). (**C**) Co-expression of DESI2 and IP10 activated caspase-3 both in CT26 and LL2 cells. CT26 and LL2 cells were treated as described in A. The treated cells were then lysed and caspase 3 activity was measured using an assay kit (*n* = 3; *p < 0.05; **p < 0.01; ^#^*p <* 0.01). (**D**) Co-expression of DESI2 and IP10 resulted in cleavage of caspase-3. CT26 and LL2 cells were subjected to the indicated treatments as described above. Caspase activation was analyzed by western blotting. β-actin was used as a loading control.

The amounts of sub-G1 cells were used to estimate cell apoptosis. Flow cytometry results showed that the apoptosis rate of CT26 cells in pDESI2-, pIP10, pVITRO2-treated and control group is 39.3%, 33.4%, 17.4% and 5.5%, respectively, whereas that of CT26 cells in pDESI2/IP10-treated group is 57.2% (Figure 2A). Similar results were also observed in LL2 cells (Figure 2A). Hoechst 33258 staining was also used to detect the apoptotic nuclear morphology in CT26 cells. As shown in Figure 2B, no condensed nuclei were observed in untreated- and pVITRO2-treated groups. However, there were some condensed or fragmented nuclei in pDESI2-, pIP10- and pDESI2/IP10-treated groups. Notably, the number of apoptotic nuclei in pDESI2/IP10-treated cells is more than that in pDESI2- or pIP10-treated cells. In addition, either DESI2 or IP10 caused caspase-3 activation, however, co-expression of DESI2 and IP10 further enhanced the caspase-3 activity (Figure 2C). The cleavage of caspase-3 were also observed in both CT26 and LL2 cells in DESI2 and/or IP10 groups (Figure 2D).

### Co-expression of DESI2 and IP10 inhibits tumor growth *in vivo*

**Figure 3:**
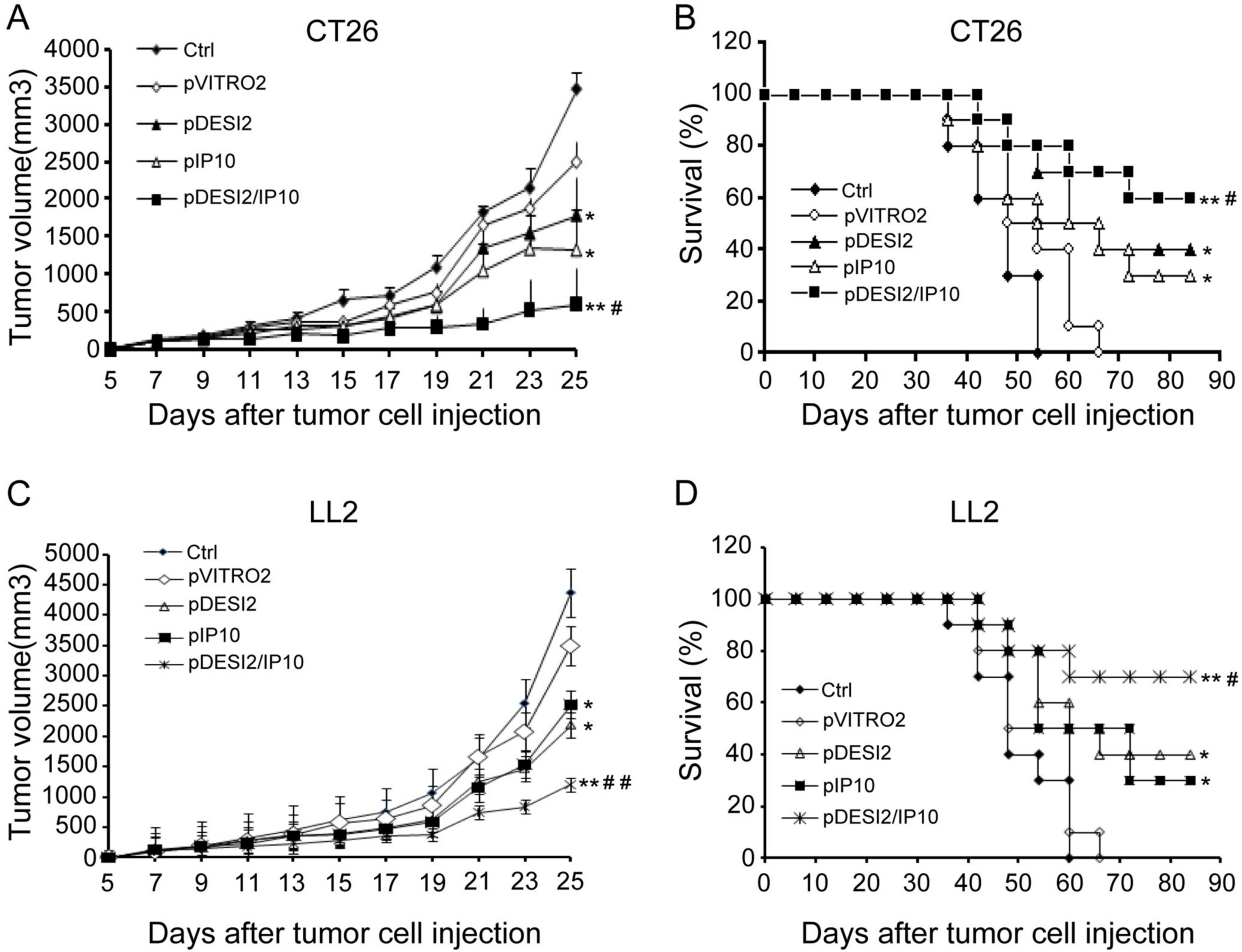
Improved therapeutic effect of co-expression of DESI2 and IP10 on two murine tumor models Female mice at 6–8 weeks of age were transplanted subcutaneously with 5 × 10^5^ CT26 or 5 × 10^5^ LL2 cells. 5 days after tumor cells were transplanted, the mice were assigned randomly to five groups and treated with 5% GS, pVITRO2, pDESI2, pIP10, pDESI2/IP10. (**A**) Suppression of tumor growth in CT26-bearing mice. The sizes (mm^3^) of tumors were monitored and recorded. pDESI2/IP10 treatment resulted in significant tumor growth inhibition. Significant differences for tumors treated with DESI2 or IP10 versus 5% GS and pVITRO2 controls (*P < 0.05; **P < 0.01); and significant difference for DESI2 plus IP10 therapy versus DESI2 or IP10 monotherapy (^#^*P <* 0.05). Bars, SD; Points, mean. (**B**) Survival curve of CT26-bearing mice per treatment group. Statistically significant differences compared with 5% GS and pVITRO2 controls (*P < 0.05; **P < 0.01). Significant differences compared with the two single-treatment groups (^#^*P <* 0.05). (**C**) Suppression of tumor growth in LL2-bearing mice. The sizes (mm^3^) of tumors were monitored and recorded. pDESI2/IP10 treatment resulted in significant tumor growth inhibition. Significant differences for tumors treated with DESI2 or IP10 versus 5% GS and pVITRO2 controls (*P < 0.05; **P < 0.01); and significant difference for DESI2 plus IP10 therapy versus DESI2 or IP10 monotherapy (^##^*P <* 0.01). Bars, SD; Points, mean. (**D**) Survival curve of LL2-bearing mice per treatment group. Statistically significant differences compared with 5% GS and pVITRO2 controls (*P < 0.05; **P < 0.01). Significant differences compared with the two single-treatment groups (^#^*P <* 0.05).

On the basis of the *in vitro* anti-tumor effects of DESI2 and/or IP10, we further investigated their antineoplastic effect *in vivo*. We found that, in both CT26 and LL2 tumor models, either DESI2 or IP10 significantly inhibited tumor growth (Figure 3A and 3C). However, DESI2 combined with IP10 had a better antitumor effect (Figure 3A and 3C).

Furthermore, the mean survival periods of CT26 tumor-bearing mice in the two control (PBS and pVITRO2) groups, pDESI2 or pIP10 group is less than 48 days, 60 and 72 days, respectively, while that of pDESI2/IP10 group is 88 days. Similar results were obtained in the other tumor model (Figure 3D). Additionally, we did not observed any significant side-effects such as weight loss, changes of behavioral habits, etc., and apparently pathological changes including heart, lung, spleen, liver and kidney (data not shown). These results clearly showed that co-expression of DESI2 and IP10 significantly improved antitumor efficacy *in vivo*.

### Co-expression of DESI2 and IP10 inhibits cell proliferation via apoptosis *in vivo*

**Figure 4:**
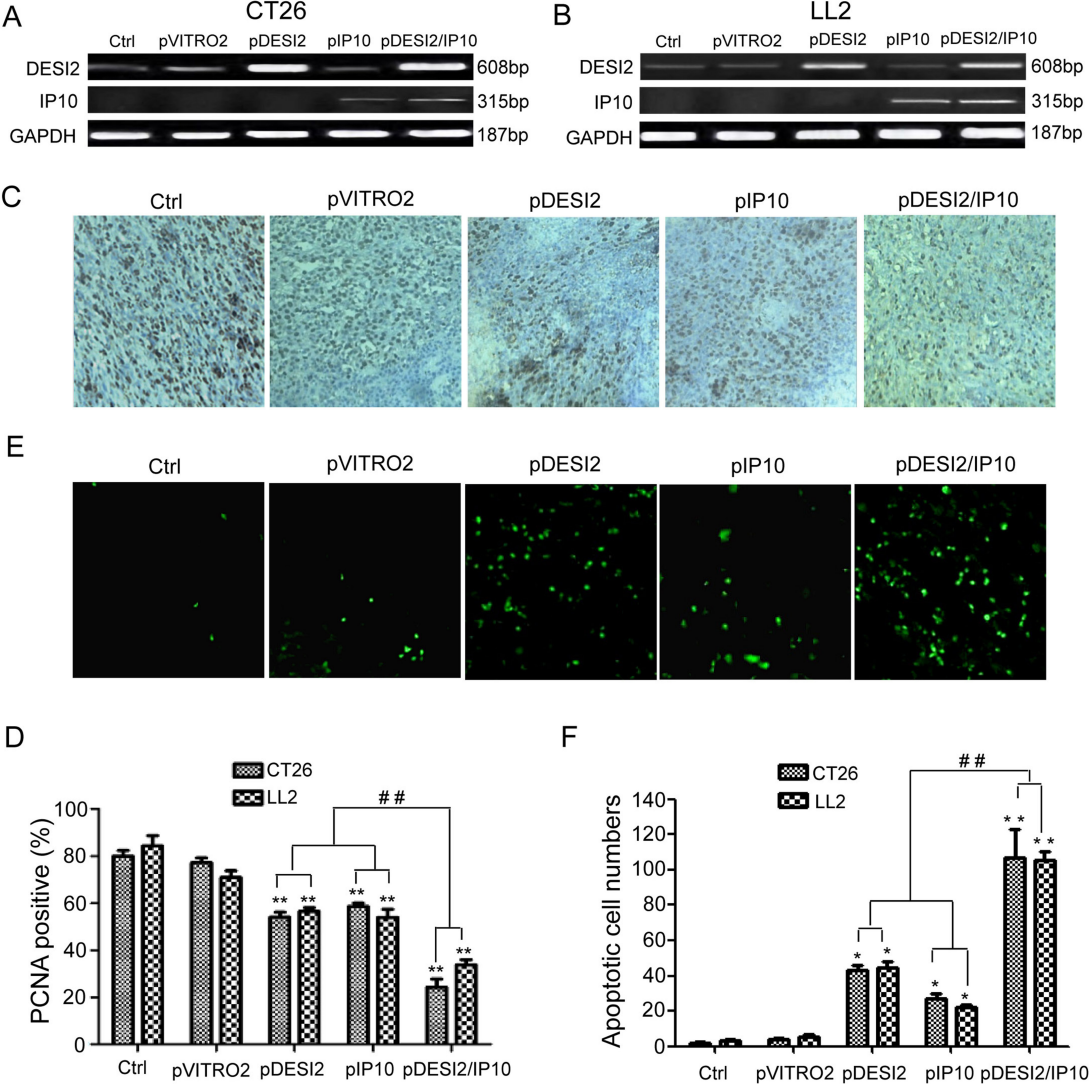
Inhibition of proliferation of tumor cells via apoptosis *in vivo* by DESI2 and/or IP10 (**A**) RT-PCR analysis of expression of exogenous DESI2 and/or IP10 in LL2 tumor tissues. When mice were killed at the end of gene therapy-related experiments, tumor tissues from one mouse, which was randomly taken out from each group, were collected for detecting DESI2/IP10 expression by RT-PCR. (**B**) RT-PCR analysis of expression of exogenous DESI2 and/or IP10 in CT26 tumor tissues. (**C**) PCNA staining of tumor tissues. Representative sections were taken from CT26 tumor tissues of mice receiving 5% GS, pVITRO2, pDESI2, pIP10, pDESI2/IP10 (original magnification, ×400). (**D**) PCNA-labeling index within CT26 and LL2 tumor tissues were estimated as the percentage of positive nuclear staining in the total number of neoplastic cells counted. Statistically significant difference in the number of PCNA-positive cells for tumors treated with DESI2 or IP10 versus 5% GS and pVITRO2 controls (**P < 0.01); significant difference for tumors treated with pDESI2/IP10 versus the two controls (**P < 0.001); and significant difference for the combination therapy versus DESI2 or IP10 monotherapy (^##^*P <* 0.01). Bars, SD; columns, mean (*n* = 3). (**E**) TUNEL staining of tumor tissues. Representative sections were taken from CT26 tumor tissues of mice receiving 5% GS, pVITRO2, pDESI2, pIP10, pDESI2/IP10 (original magnification, ×400). (**F**) Apoptotic index within CT26 and LL2 tumor tissues were counted. Statistically significant difference in the apoptotic index for tumors treated with DESI2 or IP10 versus 5% GS and pVITRO2 controls (*P < 0.05); significant difference for tumors treated with pDESI2/IP10 versus the two controls (**P < 0.01); and significant difference for the combination therapy versus DESI2 or IP10 monotherapy (^##^*P <* 0.01). The apoptotic index was calculated as a ratio of the apoptotic cell number to the total cell number in each field. Bars, SD; columns, mean (*n* = 3).

The observations that co-expression of DESI2 and IP10 displays improved antitumor efficacy *in vivo* raised a question if DESI2 and/or IP10 gene actually expressed in the tumor tissues. To confirm their expressions in tumor tissues, RT-PCR was carried out. As expected, RT-PCR results clearly showed that exogenous DESI2 and/or IP10 were overexpressed in CT26 and LL2 tumor tissues (Figure 4A and 4B), indicating that liposomal delivery of exogenous DESI2 and/or IP10 genes indeed arrived and expressed within the tumor tissues.

PCNA immunostaining was used to examine cell proliferation in tumor tissues. We observed that there were fewer PCNA-positive cells (brown) in tumor tissues from DESI2 or IP10 group mice than those in control groups (Figure 4C). However, the number of PCNA-positive cells in the pDESI2/IP10 group was the least (Figure 4D). TUNEL was further conducted to detect apoptosis of tumors. As shown in Figure 4E, there were more apoptotic cells in tumor tissue of pDESI2-treated mice than those of the two control (Glucose and pVITRO2) groups. However, the number of apoptotic cells in tumor tissue of the pDESI2/IP10 group was the largest (Figure 4E). The apoptotic index of tumor tissues from different groups were similar to the observations above, i.e., the apoptotic index of DESI2 or IP10 monotherapy group was higher than that of glucose or pVITRO2 control group. However, the apoptotic index of DESI2 plus IP10 group was the largest (Figure 4F). These results indicated that IP10 could enhance the apoptotic effect induced by DESI2.

### Co-expression of DESI2 and IP10 inhibits angiogenesis *in vivo*

**Figure 5:**
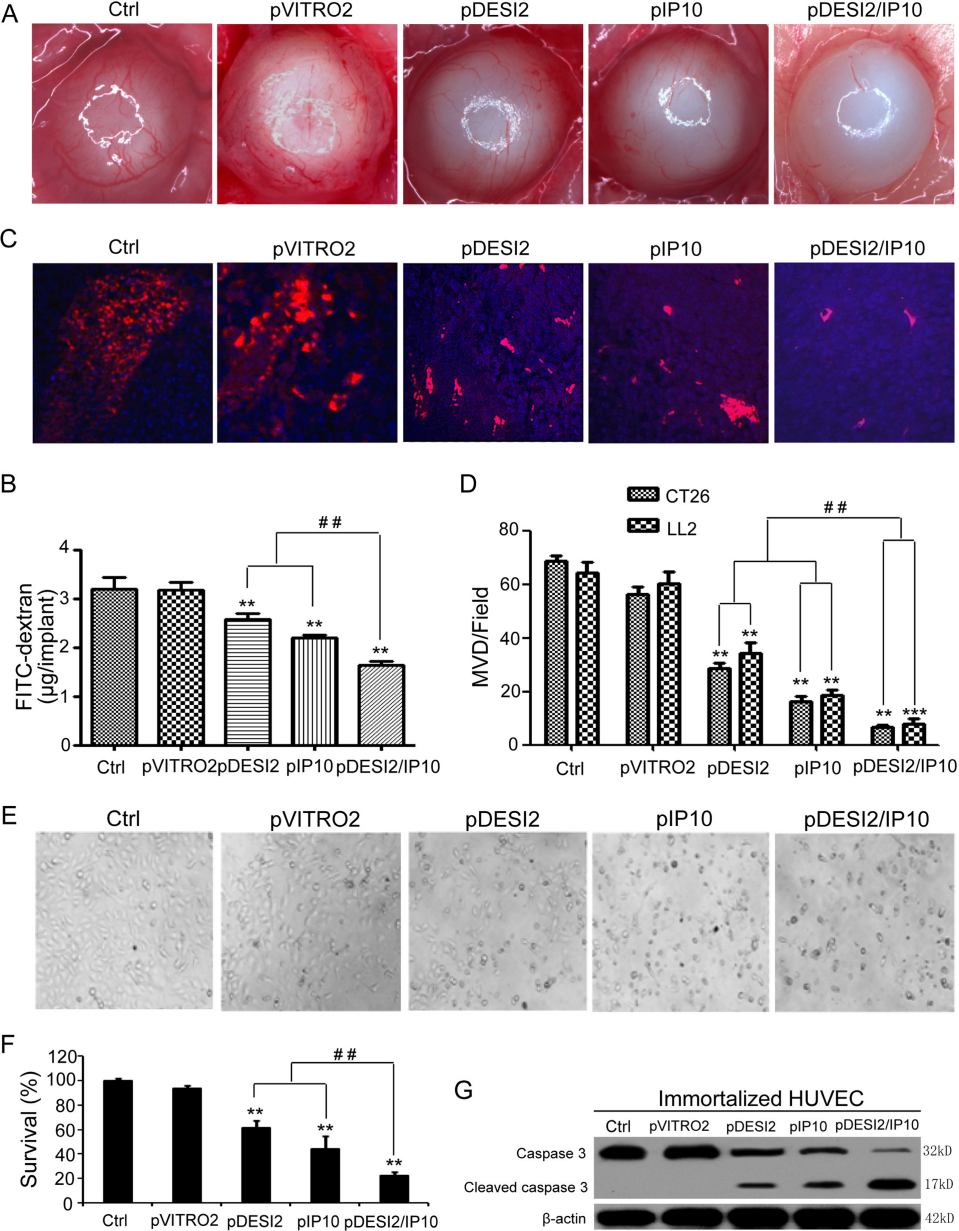
Antiangiogenesis by alginate bead assay *in vivo* (**A**) Antiangiogenesis assay by alginate bead *in vivo*. Alginate beads containing 1 × 10^5^ CT26 tumor cells were subcutaneously implanted into the backs of BALB/c mice (four beads per mouse). The treatments of mice (five mice/group) and the quantification of FITC-dextran uptake were described in Materials and Methods. Representative images of alginate beads after treatment with 5% GS, pVITRO2, pDESI2, pIP10, and pDESI2/IP10 were shown under a dissecting microscope (×10). (**B**) FITC-dextran of alginate beads was quantified. Statistically significant difference in the FITC-dextran uptake from the mice treated with IP10 or DESI2 versus 5% GS and pVITRO2 controls (**P < 0.01); significant difference in the FITC-dextran uptake from the mice treated with pDESI2/IP10 versus the two controls (**P < 0.01); and significant difference for the combination therapy versus DESI2 or IP10 monotherapy (^##^*P* < 0.01). (**C**) Frozen tumor sections from the mice treated with 5% GS, pVITRO2, pDESI2, pIP10, and pDESI2/IP10 are used to analyze the tumor angiogenesis by immunofluorescence staining with anti-CD31 antibody. Representative images of CD31 staining sections of CT26 tumor tissues were shown. (**D**) Microvessels in CT26 and LL2 tumor sections were counted in blindly chosen random fields to record microvessel density (MVD). Significant difference in MVD for tumors treated with IP10 or DESI2 versus 5% GS and pVITRO2 controls (**P < 0.01; ***P < 0.001); significant difference for tumors treated with pDESI2/IP10 versus 5% GS and pVITRO2 controls (**P < 0.01; ***P < 0.001); and significant difference for the combination therapy versus DESI2 or IP10 monotherapy (^##^*P* < 0.01). (**E**) The morphological change of immortalized HUVEC cells. The cells were untreated or transfected with pVITRO2, pDESI2, pIP10, and pDESI2/IP10 plasmids for 48 h, then used for photograph by a microscope. (**F**) Co-expression of DESI2 and IP10 resulted in significantly proliferation inhibition of immortalized HUVEC cells. The immortalized HUVEC cells were treated as the same condition described in E, then used for MTT assay. The MTT assay was carried out as described in Materials and Methods. Statistically significant differences compared with the two control groups (**P < 0.01) and the two single-treatment groups (^##^*P <* 0.01). Percentage of survival was calculated. Bars, SD; columns, mean (*n* = 3). In each experiment, the medium-only treatment (untreated) indicates 100% cell viability. (**G**) Co-expression of DESI2 and IP10 resulted in cleavage of caspase-3. The immortalized HUVEC cells were subjected to the indicated treatments as described in E. Caspase activation was analyzed by western blotting. β-actin was used as a loading control.

Alginate-encapsulated tumor cell assay was carried out to reflect angiogenesis. Representative pictures of tumor vasculature in CT26-encapsuled alginate were shown in Figure 5A, the tumors of the mice in both glucose and pVITRO2 groups showed some irregular vascular plexus, which were rich and like a ladder, while tumors from pDESI2- or pIP10-treated group displayed fewer amount of vascularization. However, the amount of tumor vasculature from pDESI2/IP10 group was the least. To further quantify the angiogenesis, FITC–dextran uptake of alginate beads was measured. The results of measurement showed that, compared with the two controls (glucose or pVITRO2 groups), the amounts of FITC-dextran uptake of IP10 and DESI2 group were significantly reduced. However, FITC-dextran uptake in DESI2/IP10 group was further reduced, and the amount of FITC-dextran uptake was the least (Figure 5B).

Microvessel density (MVD) analysis and quantification were also used to estimate tumor angiogenesis. Consistent with the results of alginate-encapsulated tumor cell assay, MVD assay and quantification results demonstrated that either DESI2 or IP10 significantly reduced the MVD of tumors from glucose- or pVITRO2-treated group, while DESI2 combined with IP10 further reduced the MVD (Figure 5C and 5D).

We further explored the possible mechanism for the antiangiogenesis of DESI2 and/or IP10, using immortalized HUVEC cells as a model. Both cell morphological change (Figure 5E) and MTT assay (Figure 5F) showed that co-expression of DESI2 and IP10 inhibited cell proliferation more effectively than that of DESI2 or IP10 alone. To investigate whether apoptosis of immortalized HUVEC cells contributes to the antiangiogenesis by DESI2 and/or IP10, apoptosis was assessed by detecting the cleavages of caspase-3. As shown in Figure 5G, either DESI2 or IP10 caused caspase-3 activation, and co-expression of DESI2 and IP10 further enhanced the activation of the caspase-3. Based on these results we speculated that inhibition of angiogenesis of DESI2 and/or IP10 are associated with the apoptosis of vascular endothelial cells.

### Immunological stimulating properties of co-expression of DESI2 and IP10 *in vivo*

**Figure 6:**
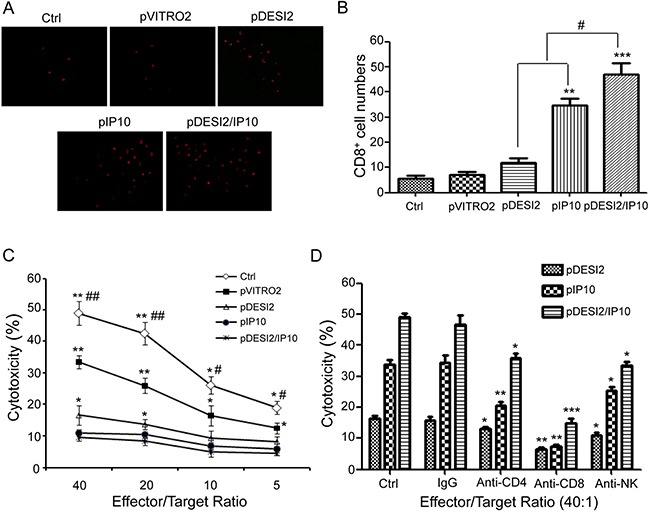
Immunological stimulating properties of DESI2 and/or IP10 (**A**) Fluorescence staining of infiltrated lymphocytes *in vivo*. The frozen sections were stained with anti-CD8+ (Cy5PE conjugate, red) antibody. (**B**) CD8+ cytotoxic lymphocyte infiltration was significantly enhanced in the tumor tissues of pDESI2/IP10, pDESI2 or pIP10 treated groups. Significant difference in CD8+T lymphocyte infiltration in tumors treated with IP10 or DESI2 versus 5% GS and pVITRO2 controls (**P < 0.01; ***P < 0.001); significant difference for tumors treated with pDESI2/IP10 versus 5% GS and pVITRO2 controls (*P < 0.05; **P < 0.01); and significant difference for the combination therapy versus DESI2 or IP10 monotherapy (^#^*P* < 0.05). (**C**) CTL-mediated cytotoxicity *in vitro*. The specific CTL activity was measured by ^51^Cr release assay. Spleen T lymphocytes derived from mice treated with pDESI2/IP10 showed higher cytotoxicity against parental CT26 cells than those from the other groups. Significant difference in CTL-mediated cytotoxicity from IP10 or DESI2 group versus 5% GS and pVITRO2 controls (*P < 0.05; **P < 0.01); significant difference in CTL-mediated cytotoxicity from pDESI2/IP10 group versus 5% GS and pVITRO2 controls (*P < 0.05; **P < 0.01); and significant difference for pDESI2/IP10 group versus DESI2 or IP10 monotherapy (^#^*P* < 0.05; ^##^*P* < 0.01). Bars, ± SD. Points, mean (*n* = 3). (**D**) Abrogation of CTL-mediated cytotoxicity *in vitro*. CTL-mediated cytotoxicity is abrogated by certain kinds of mAbs as described in Materials and Methods. The ratio of effector: target was 40:1. The pDESI2/IP10 induced tumor cytotoxic activity can be blocked by anti-CD8 mAb versus control (***P < 0.001), partial blocked by anti-CD4 mAb (*P < 0.05) or anti-NK mAb (*P < 0.05). The pIP10 or pDESI2-induced tumor cytotoxic activity was also blocked by anti-CD8 mAb (**P < 0.01), and partial blocked by anti-CD4 mAb (*P < 0.05; **P < 0.01) or anti-NK mAb (*P <0.05). Bars, SD; columns, mean (*n* = 3).

Immunofluorescence staining for CD8 was used to detect whether CD8+ T lymphocytes (CTLs) have been infiltrated into the tumors under the therapeutic condition. As shown in Figure 6A and 6B, IP10 monotherapy promoted CD8 (+) T lymphocytes to infiltrate into the tumors, while the combination of DESI2 and IP10 further enhanced CD8 (+) CTL infiltration. To investigate whether co-expression of DESI2 and IP10 could increase the CTL activity, spleen T lymphocytes were first isolated then incubated with parental CT26 cells. As expected, the cytotoxicity of spleen T lymphocytes from pDESI2/IP10 treated mice was the strongest among those of spleen T lymphocytes from other group mice (Figure 6C). Furthermore, the cytotoxicity was blocked by CD8 monoclonal antibodies, and partially abrogated by CD4 or NK monoclonal antibodies (Figure 6D). These observations indicated that an immunological stimulating function would contribute to the antitumor effects of the combination of DESI2 and IP10.

## DISCUSSION

Recently, cancer biotherapies such as tumor suppressor gene therapy, anti-angiogenesis and immunotherapy have been developed to be the fourth treatment regimen after chemotherapy, radiotherapy and surgery [34]. Previous studies have shown that DESI2 is a pro-apoptotic gene [7, 13, 14], and that it significantly induces apoptosis when overexpressed in some types of cancer cells [9, 11–15]. Several lines of evidence showed that IP10 displays anti-tumor activity by stimulating immunology, inhibiting angiogenesis and inducing apoptosis [2, 22, 29, 35]. Based on these observations, we hypothesized that DOTAP/ cholesterol cationic liposome-encapsulated recombination plasmid expressing both DESI2 and IP10 could significantly improved the therapeutic efficacy against murine carcinoma through a mechanism involving pro-apoptosis, anti-angiogenesis and immunological stimulating activities.

*In vitro*, either DESI2 or IP10 suppressed proliferation via apoptosis. DESI2 combined with IP10 further enhanced the apoptosis in colorectal cancer and lung adenocarcinoma cancer cells, as indicated by MTT (Figure 1C), colony-formation assays (Figure 1D–1F), flow cytometric analysis (Figure 2A) and Hoechst 33258 staining (Figure 2B). Apoptosis is a process composed of several signaling pathways including intrinsically mitochondrial pathway and extrinsically death-receptor pathways [9, 13]. Our previous studies have shown that DESI2 inhibits proliferation by arresting cancer cells at S phase and by inducing mitochondrial dysfunction- mediated apoptosis [7, 9, 12]. Similarly, we found that DESI2 caused activation of caspase-3 in CT26 and LL2 cells (Figure 2D).

Previous studies have shown that IP10 exerts activities of chemotaxis, pro-apoptosis, and angiostasis through binding to CXCR3 receptor [22, 26], which includes CXCR3-A, CXCR3-B and CXCR3-alt [26]. CXCR3-A is involved in cellular activation as well as angiogenesis, while CXCR3-B is associated with inhibition of cell proliferation *via* apoptosis [22, 36]. CXCR3-alt is often expressed along with CXCR3-A, and is believed to be unrelated to cell growth [26, 37]. CXCR3-B is involved in regulating the antiproliferative activity of IP10 and inhibiting endothelial cell proliferation and migration [26]. Some types of cancers such as glioblastoma, colorectal and lung cancer express CXCR3-B [26, 38]. IP10 selectively induces apoptosis of HUVEC cells because of the high ratio of CXCR3-B to CXCR3-A in endothelial cells [36]. From these observations, we assume that inhibition of proliferation by IP10 in CT26 and LL2 cells (Figure 1) and immortalized HUVEC cells (Figure 5E and 5F) may be associated with the expression of CXCR3-B. Additionally, IP10 induces apoptosis under varied conditions [26]. Sui et al. found that IP10-induced apoptosis of fetal neurons was associated with increase of mitochondrial Ca^2+^ uptake, which subsequently promoted cytochrome C release and activation of caspase-9 and -3 [26, 39], suggesting an important role of caspase-3 activation in IP10-mediated apoptosis. Similarly, IP10 also results in cleavage of caspase-3 in CT26 and LL2 cells (Figure 2D) and immortalized HUVEC cells (Figure 5G).

Consistent with the enhanced tumor suppressive activity *in vitro*, DESI2 combined with IP10 showed a significantly improved antitumor efficacy *in vivo* (Figure 3). Furthermore, the improved antitumor efficacy resulted from the augmented apoptosis, the enhanced anti- angiogenesis and the enlarged CTL effects. The augmented inhibition of proliferation via apoptosis was verified by PCNA staining (Figure 4C and 4D) and TUNEL analysis (Figure 4E and 4F), and the enhanced anti-angiogenesis was confirmed by the alginate-encapsulated tumor cell assay and CD31 immunohistochemistry staining (Figure 5), whereas the improved immunological properties was verified by the increase of the infiltration of lymphocytes (Figure 6A and 6B) and *in vitro* CTL activity (Figure 6C).

Glu-Leu-Arg (ELR) motif is critical for the function of CXC chemokines [26]. ELR-negative IP10 displays angiostatic activity [26]. Previous studies about malaria patients revealed a negative correlation of IP10 with VEGF and PDGF [26, 40, 41]. Other reports also showed that IP10 suppresses angiogenesis of tumors through antagonizing the functions of bFGF and VEGF [26, 42, 43]. In this work, we found that IP10 inhibits proliferation of immortalized HUVEC cells via apoptosis (Figure 5E and 5G). Therefore, we speculate that the enhanced anti-angiogenesis *in vivo* may, at least partly, result from IP10/DESI2-induced apoptosis of HUVEC cells.

Previous studies have shown that IP10 chemoattracts CXCR3-positive lymphocytes to tumor areas, and promotes activation of CD4+ Th and CD8+ Tc lymphocytes [22, 26, 44, 45]. Mitchell et al showed that IP10 stimulates immune response against antigens [46, 47]. Meanwhile, Hong et al observed that monocyte-derived mature DCs (mDC) displayed a strong CTL response after exposure to CCL21 chemokine [47]. Furthermore, CCL21 promoted mDC to secrete IP10, which in turn enhanced the CTL response [47]. In addition, IP10 can activate NK cells, which can kill dormant tumor cells resisting CTL-mediated lysis [48]. Similarly, we found that co-expression of DESI2 and IP10 resulted in significant increases of CD8+ T cells infiltration as well as CTL-mediated kill activity (Figure 6A and 6B). The increased CTL activities are prevented by CD8 monoclonal antibodies, and partly abrogated by CD4 or NK monoclonal antibodies, indicating its killing activity against tumor may be associated with the activation of CD4+T, CD8+ T and NK cells (Figure 6C and 6D). These data indicate that an immunological stimulating function involving the infiltration of activated T lymphocytes and activation of NK cells would contribute to the antitumor effects of the combination of DESI2 and IP10.

In summary, our data showed that co-expression of DESI2 and IP10 could effectively suppress tumor cells through inducing apoptosis, suppressing angiogenesis and triggering a CTL response. Our results may also provide a new therapeutic strategy for treating cancer.

## MATERIALS AND METHODS

### Plasmid construction

pVITRO2 (Invitrogen, San Diego, CA, USA), a dual promoter plasmid, was utilized to express both DESI2 and IP10 gene. Total RNA was isolated from cultured LL/2 murine Lewis lung carcinoma cells using Trizol reagent (Invitrogen) according to manufacturer's protocol. The open reading frame of DESI2 was cloned by RT-PCR. pBLAST49-mIP10 was purchased from InvivoGen (InvivoGen, San Diego, CA, USA). The following PCR primers were used: DESI2-forward, 5′-ATAGGATCCATGGGGGCTAACCAGTTAGT-3′; DESI2-reverse, 5′-GATAGTCGACTTA TAGTTTAGTG TGGCGCC-3′; IP10-forward, 5′-GAGGAATTCATGAAT CAAACTGCCATTCTG-3′; IP10-reverse, 5′-CAGGC TAGCTTAAG GAGATCTTTTAGACCTTTCC-3′. The incorporated 5′-BamHI, 3′-SalI, 5′-EcoRI and 3′-NheI restriction sites are shown in bold while protective base in italics. The amplified products were subcloned into pVITRO2 expression vector to generate pVITRO2-DESI2 (pDESI2)/pVITRO2 -IP10 (pIP10)/ pVITRO2-DESI2/IP10 (pDESI2 -IP10) plasmids. All the sequences were determined by DNA sequencing. An empty pVITRO2 (pEV) vector was used as a control. The plasmids were purified using Qiagen Endo-free Giga kit (Qiagen, Hilden, Germany) following the instructions of the manufacturer.

### Cell culture and transfection

Murine colon carcinoma CT26 cells, human ovarian cancer SKOV3 cells and lung adenocarcinoma cancer A549 cells were grown in RPMI-1640(GIBCO) supplemented with 10% fetal bovine serum (FBS) in humidified incubator at 37°C with 5% CO_2_. Murine Lewis lung carcinoma LL2 cells and immortalized human umbilical vein endothelial cells (HUVEC) were cultured in DMEM (GIBCO) with 10% FBS.

Transfection was performed with Lipofectamine^TM^ 2000 reagent according to the manufacturer's instruction. Briefly, cancer cells were seeded at a density of 2 × 10^5^/ 2 × 10^3^ per well in a 6/96-well plates in triplicate and incubated overnight to 70% confluence. DNA (pVITRO2, pIP10, pDESI2, pDESI2/IP10)/lipofectamine^TM^ 2000 (5 μl/ml) were complexed in DMEM/RPMI 1640 medium, and left at room temperature for 30 min. LL2, CT26, A549 and SKOV3 cells were incubated for 4 h with the above complexes, followed by rinsing 3 times, and then 1.5 ml/100 μl of DMEM/RPMI 1640 supplemented with fetal calf serum were added to each well of 6/96-well plates and incubated for further 48h.

### Treatments of cells in the *in vitro* experiments

LL2, CT26, A549 and SKOV3 cells were classified into the following five groups, and the treatments of the cells were as follows: control, the cells were left untreated, and when cultured for 72 h, cells were harvested for subsequent experiments. pVITRO2 (empty vector), the cells were first incubated for 24 h, then transfected with pVITRO2 plasmid. About 48 h after transfection, cells were harvested for subsequent experiments. pDESI2, when the cells were first incubated for 24 h, then transfected with pDESI2 plasmid. About 48 h after transfection, cells were harvested for subsequent experiments. pIP10, when the cells were first incubated for 24 h, then transfected with pIP10 plasmid. About 48 h after transfection, cells were harvested for subsequent experiments. pDESI2/IP10 (combination), when the cells were first incubated for 24 h, then transfected with pDESI2/IP10 plasmid. About 48 h after transfection, cells were harvested for subsequent experiments.

The harvested cells above were used for the following *in vitro* experiments including MTT assay, colony-formation assays, flow cytometric analysis, morphological analysis, Hoechst staining and western blot. In addition, immortalized HUVEC cells were also classified into five groups and treated as describe above. The HUVEC cells after treatments were used for MTT assay and western blot.

### Detection of DESI2/IP10 expression *in vitro* and *in vivo*

For detection of DESI2 expression *in vitro*, CT26 cells were treated according to the schedules described above. The harvested cells were used for the detection of protein expression by western blot analysis using Anti-PPPDE1/DESI2 Antibody (Everest Biotech, Ltd) and Anti-IP10 Antibody (ABcam). For detection of DESI2/IP10 expression *in vivo*, when mice were killed at the end of gene therapy experiment, tumor tissues from one mouse, which was randomly taken out from each group, were collected and used to isolate total RNA using Trizol reagent. After isolation, RNA samples were used to detect the expressions of DESI2/IP10 by RT-PCR. The primers used for PCR amplification were as follows: DESI2 (608 bp) forward, 5′-GCGGATCCGCCACCATGGC CAACCAGCCCATCATC-3′, DESI2 reverse 5′-CCGC TCGAGCTATAGTTTTGTGTGGCGCCCAGG-3′; IP10 (315 bp) forward, 5′-GAGGAATTCATG AATCAAAC TGCCATTCTG-3′; IP10 reverse, 5′-CAGGCTAGCT TAAGGAGATCTTTTAGAC CTTTCC-3′; *GAPDH* (187 bp) were designed as reported previously [13, 14, 49].

### MTT assay

Survival of cells after treatments was quantified using the MTT assay [50]. Briefly, cells were plated in 96-well plates at 5 × 10^3^ cells /well and incubated at 37°C in 5% CO_2_/95% humidity air for 24h, then cells were treated as described above. MTT was added to the medium (0.5 mg/mL) and incubated at 37°C for 4 hours. The resulting insoluble formazan was dissolved with DMSO and measured at 570 nm using a spectrophotometer. Data represent the average of three wells, and the experiment was repeated three times. Media-only treated cells served as the indicator of 100% cell viability.

### Colony-formation assays

Colony-formation assays were conducted as described previously [2]. Briefly, CT26 and LL2 cells transfected with the indicated plasmids for 24h were plated in triplicate at 500 cells per well in six-well plates, and cultured for 10 days. Then the treated cells were washed twice in PBS, fixed in cold methanol and stained with 2% crystal violet. After incubation at room temperature for 20 min, the six-well plates were washed twice in ddH_2_O, dried and colonies containing more than 50 cells were counted. The clone formation efficiency (CFE) was calculated according to the following formulas: CFE= (number of clones/number of cells inoculated) × 100%. All the experiments were repeated 3 times and the average values were reported.

### Flow cytometric analysis

Flow cytometric analysis was performed to identify sub-G1 cells/apoptotic cells and to measure the percentage of sub-G1 cells after PI staining in hypotonic buffer as described previously [51]. Briefly, cells were suspended in 1 ml hypotonic fluorochrome solution containing 50 μg propidium iodide/ml in 0.1% sodium citrate plus 0.1% Triton X-100, and the cells were analyzed by the use of a flow cytometer (ESP Elite, Coulter). Apoptotic cells appeared in the cell cycle distribution as cells with a DNA content less than that of G1 cells and were estimated with Listmode software.

### Hoechst 33258 staining

CT26 cells treated as described above were harvested, fixed for 20 min in 4% paraformaldehyde in PBS, and then washed in PBS twice. Cells were stained with Hoechst 33258 for 5 min and washed with PBS. Finally, apoptosis was visualized with a ZEISS fluorescence microscope (Jena, Inc.).

### Preparation of cationic liposome and liposome-DNA complex

The cationic liposome is composed of DOTAP: Chol. Liposome was preparated according to our previous methods [13]. Briefly, DOTAP and cholesterol were mixed at equimolar concentrations, then dissolved in chloroform. The mixture was evaporated to form a film, which was then hydrated in 5% glucose solution. The solution was first rotated at 50°C for 45 min in a water bath, then at 35°C for another 10 min. It was left overnight and sonicated at 50°C for 5 min. After sonication, the solution was put into a tube and heated at 50°C for 10 min. Then, the mixture was sequentially (five times at 0.2 μm and three times at 0.1 μm) extruded through polycarbonate membrane to decrease size using syringes. The size range is 100± 20 nm. The final cationic liposome was a small multilamellar liposome. Plasmids were packaged with liposome to form DNA–liposome complexes according to the previous report [2], then used for *in vivo* experiments.

### Animal tumor models and treatment

2 × 10^5^ CT26/LL2 cells were inoculated subcutaneously in the right flank of each BALB/c mice or C57BL/6 mice, respectively. When the size of tumors reached approximately 100 mm^3^ (5 days after tumor cell inoculation), mice were randomly divided into the following five groups (10 mice per group) and treated with: (a).100 μl 5% glucose (Ctrl); (b).10 μg pVITRO_2_ plasmid/50 μg liposome complexes in 100 μl glucose; (c). 10 μg pDESI2 plasmid/50 μg liposome complexes in 100 μl glucose; (d). 10 μg pIP10 plasmid/50 μg liposome complexes in 100 μl glucose; (e). 10 μg pDESI2/IP10 plasmid/50 μg liposome complexes in 100 μl glucose. The mice were treated with DNA-liposome complex by intravenous administration via the tail vein twice a week. Tumor size was monitored by measuring the longest dimension (length) and shortest dimension (width) in a 2-day interval with a dial caliper, and tumor volume was calculated by the following formula: tumor volume (mm^3^) = 0.52 × length (mm) × width (mm) × width (mm). At the end of the experiment, mice were sacrificed. The tumor tissues were collected for subsequent histological analysis. All studies involving mice including C57BL/6 and BALB/c mice were approved by the Institutional Animal Care and Treatment Committee of Sichuan University.

### Tlymphocyte cytotoxicity assay

Lymphocytes were isolated from the spleens of each treated mouse, then used for detecting cytotoxic T lymphocyte (CTL) activity [52]. Briefly, splenocytes isolated from the treated or control mice were depleted of erythrocytes with ammonium chloride Tris buffer. The specific CTL activity was measured by the ^51^Cr release assay using CT26 cells as target cells as previously described [53]. The percent of cytotoxicity was calculated by the formula: cytotoxicity (%) = [(experimental release-effector spontaneous release-Target spontaneous release)/ (Target maximum release-Target spontaneous release)] ×100%. In the cytotoxicity inhibition assays, effectors cells or CT26 cells were incubated with mAb at room temperature for 30 min, washed, and tested. The mAbs included anti-CD8 (10 μg /ml), anti-CD4 (10 μg/ml), anti-NK (10 μg /ml) antibodies (BD PharMingen). The above concentrations of mAb were effective in mediating their activity in preliminary experiments. Control cytotoxicity assay was performed in the presence of isotype IgG mAb (BD PharMingen).

### Alginate-encapsulated tumor cell assay

Alginate-encapsulated tumor cell assays were conducted as described previously with a slight modification [13, 54]. Briefly, CT26 cells were resuspended in sodium alginate solution (w/v, 1.8%) and added dropwise to 250 mM calcium chloride solution. One of the formed alginate beads contained approximately 1 × 10^5^ tumor cells. BALB/c mice were then anesthetized, and four beads were implanted subcutaneously into an incision made on the dorsal side. Mice were subdivided into five groups (five mice per group) and treated as described above. The treatments were started 24 h after the beads were implanted. Two weeks later, the mice were injected i.v. with 100 μL of 100 mg/kg FITC–dextran (Sigma) solution through the tail vein. Beads were removed and photographed 20 min after FITC–dextran injection. The FITC–dextran uptake was measured against a standard curve of FITC–dextran.

### Histological analysis

Dissected tumors were divided in half, one-half for paraffin sections fixed in 10% NBF and embedded in paraffin, and the other half frozen at −80°C. For microvessel density (MVD) analysis, frozen sections (7 μm) were fixed in acetone, incubated, and stained with an antibody reactive to CD31 as reported previously [13]. MVD was determined by counting the number of microvessels per high-power field as described previously [55, 56]. For assessment of proliferation, immunohistochemical staining of PCNA were performed. For quantitative assessment of apoptosis, TUNEL was carried out with an *In situ* Cell Death Detection Kit (Hoffmann-La Roche, Basel, Switzerland) following the manufacturer's protocol. For observations of potential side effects, the tissues (including heart, liver, spleen, lung, kidney, and brain) were fixed in 10% NBF and embedded in paraffin. Sections (3–5 μm) were stained with H&E. Sections in H&E staining and immunohistochemical staining were observed by two pathologists in a blinded manner.

### Statistical analysis

The statistical analysis was carried out using SPSS software (version 12.0 for Windows, SPSS UK Ltd., Woking, Surrey, UK). All of the values were expressed as means ± SD. Data were analyzed by one-way ANOVA, and then differences among the means were analyzed using Tukey–Kramer multiple comparison test. Survival curves were constructed according to the Kaplan–Meier method, and statistical significance was determined by the log-rank test. Differences were considered significant at *P <* 0.05.
